# Transiently lowering tumor necrosis factor-α synthesis ameliorates neuronal cell loss and cognitive impairments induced by minimal traumatic brain injury in mice

**DOI:** 10.1186/s12974-015-0237-4

**Published:** 2015-03-07

**Authors:** Renana Baratz, David Tweedie, Jia-Yi Wang, Vardit Rubovitch, Weiming Luo, Barry J Hoffer, Nigel H Greig, Chaim G Pick

**Affiliations:** Department of Anatomy and Anthropology, Sackler School of Medicine, Tel-Aviv University, Tel-Aviv, Israel; Drug Design and Development Section, Translational Gerontology Branch, Intramural Research Program, National Institute on Aging, National Institutes of Health, BRC Room 05C220, 251 Bayview Blvd., Baltimore, MD 21224 USA; Graduate Institute of Medical Sciences, College of Medicine, Taipei Medical University, Taipei, Taiwan; Department of Neurosurgery, Case Western Reserve University School of Medicine, Cleveland, OH USA

## Abstract

**Background:**

The treatment of traumatic brain injury (TBI) represents an unmet medical need, as no effective pharmacological treatment currently exists. The development of such a treatment requires a fundamental understanding of the pathophysiological mechanisms that underpin the sequelae resulting from TBI, particularly the ensuing neuronal cell death and cognitive impairments. Tumor necrosis factor-alpha (TNF-α) is a cytokine that is a master regulator of systemic and neuroinflammatory processes. TNF-α levels are reported to become rapidly elevated post TBI and, potentially, can lead to secondary neuronal damage.

**Methods:**

To elucidate the role of TNF-α in TBI, particularly as a drug target, the present study evaluated (i) time-dependent TNF-α levels and (ii) markers of apoptosis and gliosis within the brain and related these to behavioral measures of ‘well being’ and cognition in a mouse closed head 50 g weight drop mild TBI (mTBI) model in the presence and absence of post-treatment with an experimental TNF-α synthesis inhibitor, 3,6′-dithiothalidomide.

**Results:**

mTBI elevated brain TNF-α levels, which peaked at 12 h post injury and returned to baseline by 18 h. This was accompanied by a neuronal loss and an increase in astrocyte number (evaluated by neuronal nuclei (NeuN) and glial fibrillary acidic protein (GFAP) immunostaining), as well as an elevation in the apoptotic death marker BH3-interacting domain death agonist (BID) at 72 h. Selective impairments in measures of cognition, evaluated by novel object recognition and passive avoidance paradigms - without changes in well being, were evident at 7 days after injury. A single systemic treatment with the TNF-α synthesis inhibitor 3,6′-dithiothalidomide 1 h post injury prevented the mTBI-induced TNF-α elevation and fully ameliorated the neuronal loss (NeuN), elevations in astrocyte number (GFAP) and BID, and cognitive impairments. Cognitive impairments evident at 7 days after injury were prevented by treatment as late as 12 h post mTBI but were not reversed when treatment was delayed until 18 h.

**Conclusions:**

These results implicate that TNF-α in mTBI induced secondary brain damage and indicate that pharmacologically limiting the generation of TNF-α post mTBI may mitigate such damage, defining a time-dependent window of up to 12 h to achieve this reversal.

## Introduction

Traumatic brain injury (TBI) is a common cause of morbidity and mortality across both the civilian and military populations, with a reported worldwide annual incidence of some ten million cases [1]. Indeed, within the US alone, TBI accounts for some 1.7 million emergency department visits - a number that likely underestimates its true incidence [2] - and is credited with some 30% of all injury-related deaths [3]. In essence, TBI is elicited following the unexpected application of an external force to the head. Patients who survive such injury often present with persistent long-term disabilities that require rehabilitation - a costly 52 billion dollars annual expense in the US alone [4-6]. The severity of ensuing disabilities varies and often may be associated with the severity of the injury itself [7]. Mild TBI (mTBI) accounts for some 80% to 90% of cases, and arising common disabilities include sensory-motor problems, learning and memory deficits, anxiety, and depression [8,9]. Of significant additional concern, mTBI may predispose long-term survivors to age-related neurodegenerative disorders by providing a risk factor for the development of Alzheimer’s disease, Parkinson’s disease, and post-traumatic dementia [10-14], with the older people being most vulnerable [15,16]. Despite significant ongoing research and advancements in our understanding of the molecular and cellular changes that occur after TBI, no effective pharmacological treatment is currently available [17,18].

mTBI-associated brain damage can be subdivided into two phases: an initial primary phase that is immediate and results from the mechanical force(s) applied to the skull and brain at the time of impact, potentially inducing shearing and compression of neuronal and vascular tissue that results in brain contusion, axonal injury, blood vessel rupture, and hemorrhage. This is followed by an extended second phase that involves cascades of biological processes initiated at the time of injury that may persist over subsequent days, weeks, and possibly months, consequent to ischemia, neuroinflammation, glutamate toxicity, altered blood-brain barrier permeability, oxidative stress, astrocyte reactivity, cellular dysfunction, and apoptosis [19-22]. As secondary brain injury may be reversible, in order to develop an effective treatment, it is imperative to understand the biological cascades that drive the delayed secondary phase that occurs following TBI [23-25].

It is widely recognized that inflammatory cytokines, chemokines, and growth factors play significant roles in the pathophysiology of TBI. Albeit that initiation of an inflammatory response can be essential to promote neuroreparative mechanisms in response to a physiological insult [26-28], if this is excessive or unregulated, it can augment neuronal dysfunction and degeneration by inducing a self-propagating pathological cascade of neuroinflammation [29-31]. Shortly following TBI, substantial synthesis and release of proinflammatory cytokines occur from astrocytes and microglia, particularly tumor necrosis factor-α (TNF-α) with mRNA and protein levels becoming acutely elevated within as little as 17 min after injury seen in post-mortem brains from patients who died shortly after TBI [32]. A parallel rapid sequence has been described in rodent TBI animal models in which a TNF-α rise precedes the appearance of ensuing cytokines [33-35]. Depending on its signaling pathway, TNF-α can exacerbate trauma and oxidative stress within the brain and contribute to glutamate release and blood-brain barrier dysfunction that can lead to further influx of inflammatory factors from blood to brain [36].

Inhibiting the generation of TNF-α may thus reinforce its role in mTBI and define its value as a potential treatment target, as it is considered a master regulator of the inflammatory response. Sudden and substantial rises in TNF-α can induce a diverse array of cell death processes, including NF-kB activation, apoptosis, and necrosis [37]. In addition, an increase in TNF-α levels trigger glutamate release from astrocytes, which can lead to glutamate excitotoxicity [38]. Although the elevation of TNF-α levels in the early hours post TBI can be harmful [39-41], cytokine balance has been reported as essential for long-term recovery from injury [40-42]. In this current study, rather than utilizing a TNF-α antibody approach to capture and clear it before it can potentially reach its target, as is effectively achieved in the treatment of rheumatoid arthritis, the experimental drug 3,6′-dithiothalidomide was employed to reduce TNF-α synthesis [43] and thereby maintain but dramatically lower its physiological release pattern. In our previous studies, we effectively used 3,6′-dithiothalidomide to ameliorate cognitive deficit following mTBI [44]. However, our previous work did not define the therapeutic window for 3,6′-dithiothalidomide, the extended time course of TNF-α overproduction, and the histochemical changes in neurons and glia correlated with injury. We extend our previous finding in the present study, correlating the potential role of mTBI-induced TNF-α release with neuronal loss, apoptosis, and astrocyte elevation, and defining a window of opportunity for potential treatment.

## Materials and methods

### Animals

Male ICR mice (30 to 40 g of weight and 6 to 8 weeks of age) were bred and raised within the vivarium of Tel Aviv University, Israel, originally derived from breeding pairs purchased from HSD Jerusalem, Israel. They were housed four to six per cage, maintained at a constant 22 ± 1°C, had *ad libitum* access to food and water, and kept on a 12:12 h light/dark cycle. Lighting during the light phase remained constant, and all experimental manipulations were undertaken during this light phase of the cycle. A minimum number of animals were included into studies, and all efforts were made to minimize potential suffering. Each animal was used for only a single experiment, and all experimental procedures and housing conditions were approved by the Institutional Animal Care and Use Committee of Tel Aviv University (M-10-006).

### Mild traumatic brain injury

Mice were subjected to mTBI using a weight drop device that has previously been described [44-46]. Mice were anesthetized with isoflurane (Merck & Co., Inc., Whitehouse Station, NJ, USA) and then placed under the device. The weight drop apparatus comprised of a cylindrical-shaped 50-g piece of metal with a rounded spherical tip, which was dropped through a vertical metal guide tube (diameter 13 mm × length 80 cm). Anesthetized mice were carefully positioned with their head supported and immobilized by a sponge so that the right temporal side of the head, between the corner of the eye and the ear, was directly below the guide tube opening. The sponge allowed anterior/posterior motion of the head without rotational movement at the moment of impact following weight drop [44-46]. Sham mice were submitted to the same procedure as described for mTBI, but without release of the weight.

### Drug administration

Synthesis of 3,6′-dithiothalidomide (Merck & Co., Inc., Whitehouse Station, NJ, USA) was achieved by a published synthetic route [43], and chemical characterization confirmed the structure of the final product with a chemical purity of 99.8%. The agent was prepared as a suspension in 1% carboxymethyl cellulose (formulated in isotonic saline; Merck & Co., Inc., Whitehouse Station, NJ, USA) immediately prior to daily use in each study to provide a final dose of 28 mg/kg (0.1 ml/10 g) body weight. Either 3,6′-dithiothalidomide or similarly prepared vehicle was administered by the intraperitoneal (i.p.) route from 1 to 18 h post injury or sham procedure, depending on the measures evaluated (whether for ELISA, immunohistochemistry, or behavioral studies).

### TNF-α analysis by ELISA

To verify the occurrence of TNF-α elevation in our mTBI model and define its time dependence, mice were subjected to mTBI and brains were removed at specific times thereafter (1 to 18 h; *n* = 4 to 5 per time). The right cortex was immediately frozen in liquid nitrogen and homogenized with appropriate protease inhibitors (Halt Protease Inhibitor Cocktail; Sigma-Aldrich, St. Louis, MO, USA). The samples were then quantified for TNF-α levels by ELISA assay (BioLegend, San Diego, CA, USA).

### Physiological parameters of well-being

Rectal temperature was recorded with a mouse thermometer. Baseline values (°C) were evaluated 30 min before 3,6′-dithiothalidomide administration and at 1 and 4 h following mTBI or sham procedure.

Anxiety-like behavior and motor activity were evaluated by elevated plus maze. The maze was elevated 60 cm above the floor level and comprised of 4 arms (30 × 5 × 15 cm) along which mice could walk that formed a ‘+’ shape [47]. Two conjoined arms were open (without walls) and the other two were closed (with walls but no ceiling). On evaluation days, mice were placed at the center of the plus-maze, facing one of the open arms and their time spent within the open arms was recorded over a 5-min period. The maze was cleansed with 70% ethanol (ETOH; *v*/*v*) between animals.

### Cognitive behavioral tests

Two behavioral paradigms were evaluated: Y-maze and novel object recognition (NOR).

### Y-maze test

Spatial memory was evaluated by Y-maze, as initially described by Dellu and colleagues [48], and is a task that takes advantage of a preference of rodents to explore novel rather than familiar places. The Y-maze was erected from black Plexiglas and comprised of three alike arms (30 × 8 × 15 cm length, set at an angle of 120° from one another). Evaluation comprised of two trials separated by a 2-min interval (during which the mouse was returned to its home cage). The initial ‘familiarization’ trial was of 5-min duration with only two arms open (one termed the ‘start’ arm and the other the ‘old’ arm), with the third (‘novel’) arm blocked by a door. The second trial was of 2-min duration, and all three arms were open. The time spent in each of the arms was recorded, and discrimination of spatial novelty was determined as a preference index [49] calculated as (time in the novel − time in the old arm)/(time in the novel + time in the old arm). The apparatus was cleansed between trials with 70% ETOH (*v*/*v*).

### NOR test

An object recognition test to evaluate short-term recognition memory [50] was undertaken within an open field that comprised a black Plexiglas arena (59 × 59 cm size) surrounded by 20-cm black walls. The task takes advantage of a predisposition for rodents to explore new objects and included three trials of 5-min duration separated by a 24-h interval. On the initial day of evaluation, mice were individually placed within the empty arena for habituation. The following day, mice were placed into the same arena that had two identical objects, A and B, positioned 40 cm from one another and 10 cm from the walls. On the third day, mice were again placed into the arena; however, object A remained the same as the preceding day and new object C replaced prior object B. The arena and objects were thoroughly cleansed (70% ETOH *v*/*v*) between each trial. Object exploration (defined as rearing on the object or sniffing it at a distance of less than 2 cm and/or nose touching it) was recorded and discrimination of recognition novelty was determined as a preference index [49]: (time exploring the new object − time exploring the old object)/(total time exploring an object). Mice that explored objects for less than 10% of the total available time were excluded from analyses.

### Immunohistochemistry/immunofluorescence brain slice studies

A cohort of mTBI and sham mice were anesthetized at 72 h following the procedure by excess ketamine + xylazine administration and were immediately perfused transcardially with PBS followed by 4% paraformaldehyde ((PFA) in 0.1 M phosphate buffer, pH 7.4). Their brains were removed, fixed overnight (4% PFA in 0.1 M phosphate buffer, pH 7.4), and then placed in 30% sucrose for 48 h. Coronal sections (30 μm) were cut on a cryostat, placed in cryoprotectant, and stored at −20°C until use. Thereafter, 5 sections of cortex and 5 of hippocampus were blocked by incubation with 0.1% Triton X-100 in phosphate-buffered saline (PBST) and 10% normal horse serum for 1 h at 25°C. The primary antibodies, mouse anti-neuronal nuclei (NeuN; 1:50, Millipore, Danvers, MA, USA, Cat#MAB3377), mouse anti-glial fibrillary acidic protein (GFAP; 1:10,000, Millipore, Cat#MAB3402), and rabbit anti-BH3-interacting domain death agonist (BID; 1:50, Cell Signaling, Danvers, MA, USA, Cat#9942), were then dissolved in PBST and 2% normal horse serum and incubated with the sections for 48 h at 4°C. Following rinsing in PBST, sections were incubated for 1 h at 25°C with DyLight™ 594-conjugated AffinityPure Donkey Anti-rabbit IgG and DyLight™ 488-conjugated AffinityPure Donkey Anti-mouse IgG (1:300; Jackson Laboratories, Bar Harbor, ME, USA). After rinses in PBST, sections were mounted on dry gelatin-coated slides and evaluated for fluorescence with a Zeiss LSM 510 confocal microscope with × 20 and × 63 lens (Carl Zeiss, Jena, Germany). For each brain, three to five sections were taken and the average numbers of cells within the hippocampus and the temporal cortex were calculated within defined fields of either 140^2^ or 440^2^ μM. Evaluation of immunohistochemical slides for immunofluorescence was undertaken in a blinded manner, and the omission of primary antibodies was routinely undertaken in the generation of negative control sections. Analyses were performed by Imaris program for color quantification (Bitplane AG, Zurich, Switzerland).

### Data analyses

Results throughout are presented as mean ± SEM values and were analyzed by SPSS 18 software (Genius Systems, Petah Tikva, Israel). One-way ANOVAs were performed to compare between all groups, followed by least significant difference (LSD) *post hoc* tests. ANOVA-repeated measures were performed to compare rectal temperatures.

## Results

### Evaluation of well-being

‘Basic well-being,’ a concept that underlies the combined health and wellness of an animal [51], was evaluated across all mice groups and combined subjective measures, such as the grooming and appearance, righting skills, ambulation, and blinking reflex, with objective ones that included the parameters of weight, body temperature, anxiety-like behavior, and motor skills.

Subjectively and in accord with prior studies [45], mice subjected to this type of mTBI were indistinguishable from those subjected to the sham procedure when evaluated at 1 or 24 h later, irrespective of 3,6′-dithiothalidomide or vehicle administration. Rectal temperature measurements were used to monitor potential core temperature changes induced by either brain injury or 3,6′-dithiothalidomide administration, and no significant difference (NS) was found either between animal groups [*F*(2,12) = 0.084, NS] or across measurement times (30 min before injury and 1 and 4 h post-mTBI/injection) [*F*(2,12) = 3.630, NS] (data not shown).

The elevated plus maze was used to examine anxiety-like behavior and motor activity. No differences were found between any groups in anxiety-like behavior at 72 h and 7 days post-injury [*F*(5,56) = 0.791, NS] [*F*(5,47) = 0.765, NS], respectively (data not shown). Likewise, no differences were evident between any groups in relation to motor skills evaluated at 72 h and 7 days post-injury [*F*(5,56) = 1.13 NS] [*F*(5,47) = 0.798, NS], respectively (data not shown). Together these result indicate that mice were healthy and that neither mTBI nor 3,6′-dithiothalidomide impacted their well-being.

### Time-dependent changes in TNF-α levels in brain tissue

**Figure 1:**
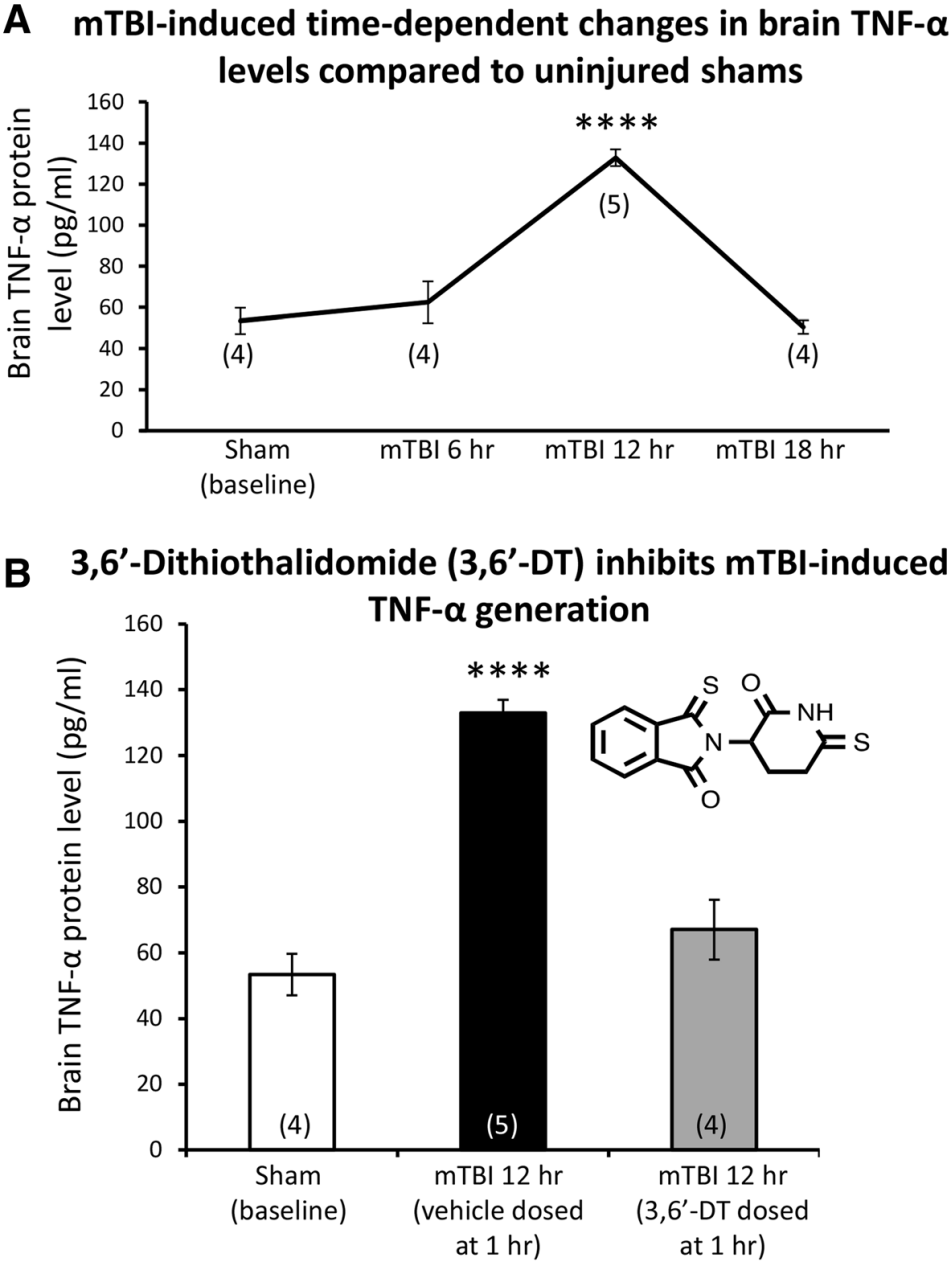
**mTBI induces a time-dependent rise in brain TNF-α** **levels.** Right (ipsilateral to mTBI) cerebral cortex protein extracts were prepared from sham or mTBI mice at the indicated time points post injury. **(A)** Time-dependent brain levels of TNF-α at baseline (sham) and post injury. At 12 h post mTBI, TNF-α levels peaked (132.8 vs. 53.4 (sham) pg/ml, *p* < 0.0001). By 18 h post injury, TNF-α levels returned to baseline (50.5 pg/ml). **(B)** Treatment with 3,6′-dithiothalidomide (3,6-DT) at 1 h after mTBI prevented the TNF-α elevation evident at 12 h post mTBI (3,6′-DT + mTBI 67.1 pg/ml vs. mTBI 132.8 pg/ml, *p* < 0.0001). In both **(A)** and **(B)**, **** was significantly different from all other groups (*p* < 0.0001).

As illustrated in Figure 1, mice challenged with mTBI demonstrated a time-dependent rise in brain protein levels of TNF-α that were increased by 2.5-fold, peaked at 12 h post injury, and returned to baseline by 18 h [*F*(3,13) = 30.529, *p* < 0.0001]. LSD *post hoc* analyses confirmed that the 12-h mTBI group was significantly different from all other groups (*p* < 0.0001). Levels were elevated to 132.9 pg/ml at 12 h versus a baseline value of 53.4 pg/ml. In animals subjected to mTBI and administered 3,6′-dithiothalidomide 1 h post injury, the elevated TNF-α 12 h post injury response was ameliorated. Specifically, mice treated with 3,6′-dithiothalidomide post injury had similar brain TNF-α levels as the sham group, 67.0 and 53.4 pg/ml, respectively, *F*(4,17) = 14.579, *p* < 0.0001, Figure 1B. LSD *post hoc* analyses confirmed that the mTBI 12-h group was significantly different from all other groups (*p* < 0.0001).

### mTBI- and treatment-induced changes in cognitive function

**Figure 2:**
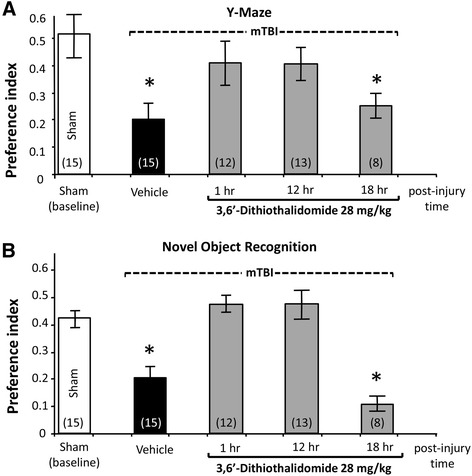
**mTBI induces impairments in performance in both a Y-maze and novel object recognition (NOR) preference index paradigms that are ameliorated by 3,6′-dithiothalidomide when administered up to 12 but not 18 h post injury. (A)** Performance of mice was quantitatively assessed in a Y-maze and **(B)** in a NOR paradigm at 7 days following mTBI as a preference index that was calculated as (time associated with the novel − time with the old arm or object)/(time with the novel + time with the old arm or object). Values are mean ± SEM values; a one-way ANOVA indicates that mTBI animals had a deficit in spatial (Y-maze) and visual (NOR) memory performance compared with all the other groups (*p < 0.05) with the exception of animals dosed with 3,6′-dithiothalidomide at 18 h post injury. No differences were found between any of the other groups (control (sham) 1 and 12 h 3,6′-dithiothalidomide dosing), suggesting complete amelioration by 3,6′-dithiothalidomide when administered within 12 h of injury.

When evaluated by Y-maze at 7 days post procedure, vehicle-treated mTBI-challenged mice demonstrated a significant impairment in spatial memory, as compared to sham control animals. This mTBI-induced deficit was ameliorated by a single dose of 3,6′-dithiothalidomide administered either 1 or 12 h post injury. However, when 3,6′-dithiothalidomide administration was withheld until 18 h, mice displayed impairment and, together with the mTBI vehicle group, their preference index was significantly reduced compared to sham controls [*F*(4,57) = 6.462, *p* < 0.01] (Figure 2A). LSD *post hoc* analyses confirmed that the mTBI + vehicle and the mTBI + 18 h 3,6′-dithiothalidomide groups were significantly different from all other groups (*p* < 0.05).

As illustrated in Figure 2B, the spatial deficit evident in vehicle-treated mTBI mice in the Y-maze was also seen with the NOR paradigm. Here too, the administration of a single dose of 3,6′-dithiothalidomide to mTBI mice 1 or 12 h following injury fully mitigated the deficit, but delaying administration to 18 h post injury did not. Specifically, the mTBI vehicle and mTBI + 18 h 3,6′-dithiothalidomide groups displayed a significantly reduced index preference versus sham controls [*F*(4,57) = 8.975, *p* < 0.001]. LSD *post hoc* analyses established that the mTBI + vehicle and the mTBI + 18 h 3,6′-dithiothalidomide groups were significantly different from all other groups (*p* < 0.05).

Together these results extend the work of Baratz and colleagues [44] and define a therapeutic window of up to 12 h post mTBI to mitigate cognitive deficits by lowering TNF-α generation, as well as documenting the time course of TNF-α elevation.

### Immunofluorescence

To evaluate the impact of mTBI at the cellular level, particularly in relation to the described amelioration of cognitive deficits imparted by lowering TNF-α generation, immunohistochemical analyses were undertaken at 72 h post injury. These focused on two key brain areas ipsilateral to injury: the cerebral cortex, as the area closest to impact, and the dentate gyrus, a region of the hippocampal formation considered to contribute to the formation of new episodic memory [52,53], the spontaneous exploration of novel environments, and other mnemonic functions [53,54].

**Figure 3:**
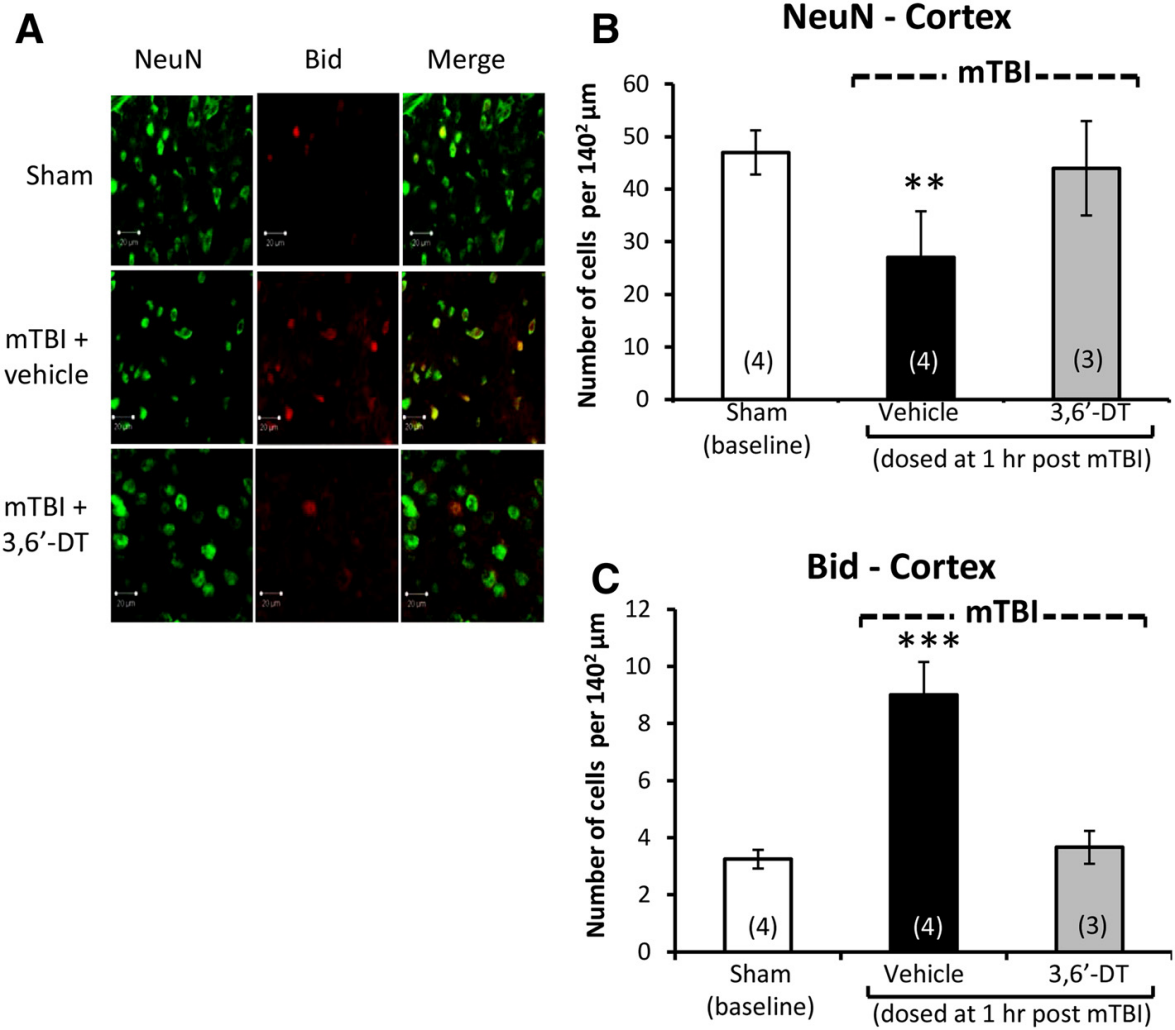
**Neuronal loss and apoptosis is induced by mTBI in cerebral cortex ipsilateral to injury and mitigated by 3,6′-dithiothalidomide.** At 72 h post injury, cerebral cortex ipsilateral to mTBI was assessed for cellular changes. **(A)** and **(B)** A decline in neuronal number indicative of neuronal loss (NeuN - green) was evident post mTBI (*p* < 0.01). Treatment with 3,6′-dithiothalidomide at 1 h post-injury prevented such a change. **(A)** and **(C)** An elevation in BID (a marker for apoptosis - red) was evident within mTBI brains (*p* < 0.001). No changes in apoptotic cell death were found in animals that were treated with 3,6′-dithiothalidomide (as compared to sham animals). Within **(A)** (representative sections within the cerebral cortex), the bar is equal to 20 μm in length.

**Figure 4:**
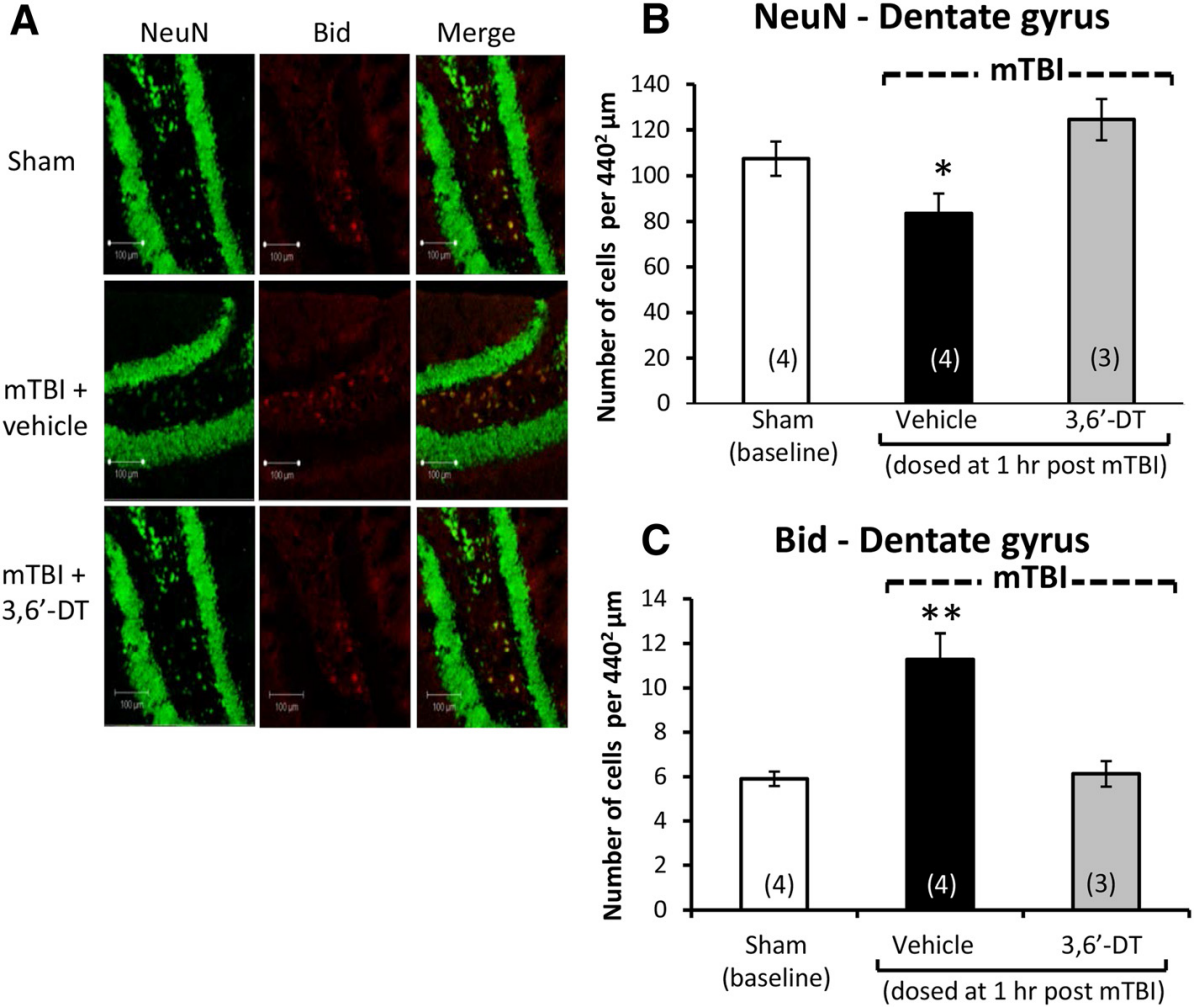
**Neuronal loss and apoptosis is induced by mTBI in the dentate gyrus ipsilateral to injury and mitigated by 3,6′-dithiothalidomide.** At 72 h post injury, the dentate gyrus of the hippocampus ipsilateral to mTBI was evaluated for cellular changes. **(A)** and **(B)** Neuronal loss (NeuN - green) was found post mTBI (*p* < 0.05). Treatment with the 3,6′-dithiothalidomide at 1 h post-injury prevented this loss. **(A)** and **(C)** An increase in BID (a marker for apoptosis in red) was evident in the mTBI brains (*p* < 0.01). No change in apoptotic cell death was apparent in animals treated with 3,6′-dithiothalidomide (as compared to sham animals). Within **(A)** (representative sections within the dentate gyrus), the bar is equal to 100 μm in length.

**Figure 5:**
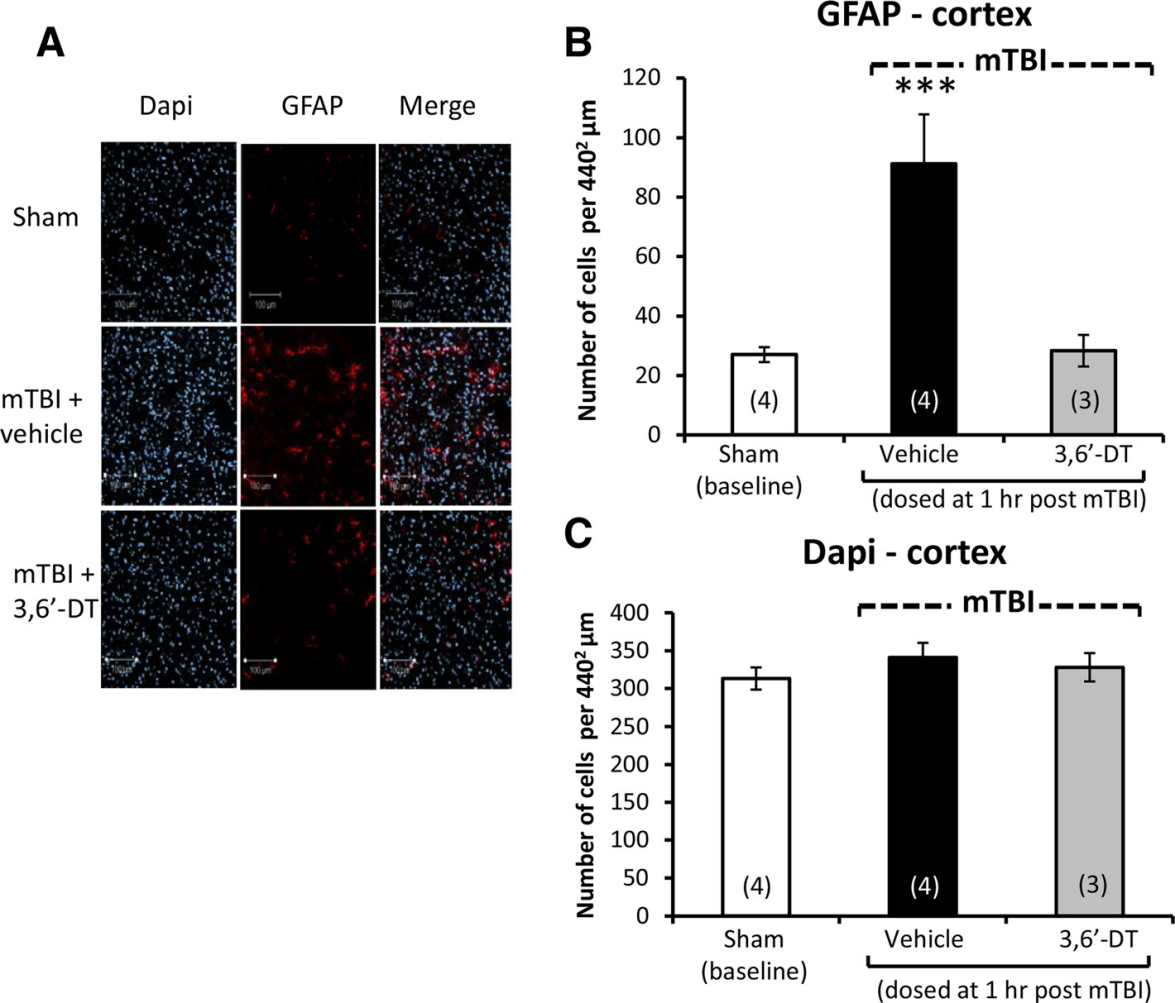
**mTBI induces an elevation in astrocyte number in ipsilateral cerebral cortex that is inhibited by 3,6′-dithiothalidomide.** At 72 h post injury, cerebral cortex ipsilateral to mTBI was assessed for cellular changes. **(A)** and **(B)** Astrocyte number (GFAP - red) was increased post mTBI (*p* < 0.001). Treatment with 3,6′-dithiothalidomide at 1 h post-injury prevented this. **(A)** and **(C)** No difference in total number of cells was evident between groups, as revealed from DAPI (blue) staining. Within **(A)** (representative sections within the cerebral cortex), the bar is equal to 100 μm in length.

**Figure 6:**
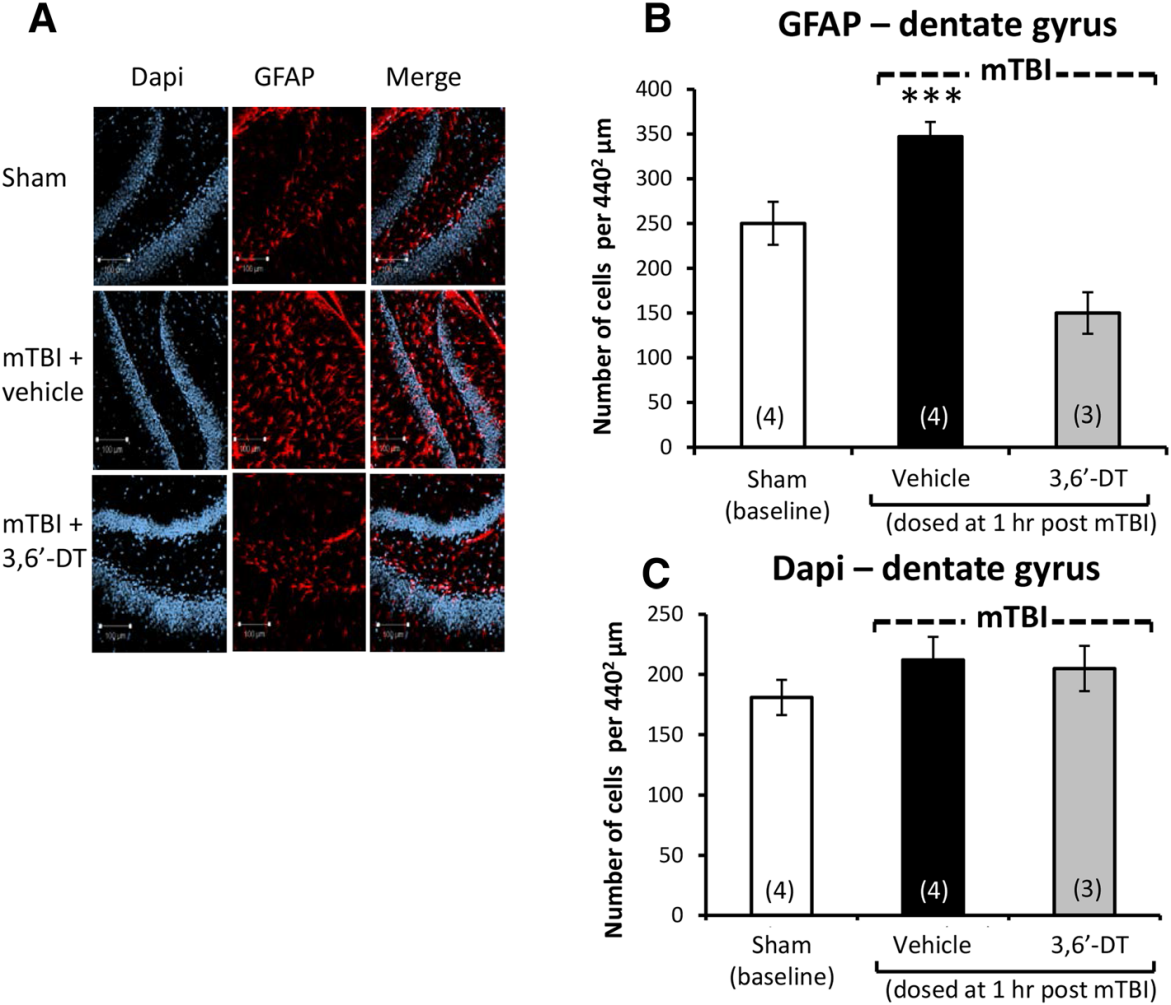
**mTBI induces an elevation in astrocyte number in ipsilateral dentate gyrus that is inhibited by 3,6′-dithiothalidomide.** At 72 h post injury, dentate gyrus ipsilateral injury was assessed for cellular changes. **(A)** and **(B)** Astrocyte number (GFAP - red) was elevated post mTBI (*p* < 0.001). Treatment with 3,6′-dithiothalidomide at 1 h post-injury inhibited this. **(A)** and **(C)** No difference in total number of cells was apparent between groups, as evaluated by DAPI (blue) staining. Within **(A)** (representative sections within the dentate gyrus), the bar is equal to 100 μm in length.

Illustrated in Figures 3A and 4A are brain regions (cerebral cortex and dentate gyrus, respectively) displaying immunofluorescence associated with (i) NeuN, a neuronal nuclear protein that is widely used as a marker of adult neurons, and with (ii) BID, a proapoptotic Bcl-2 protein. Quantification of NeuN staining revealed a neuronal loss in both the cortex [*F*(3,13) = 7.198, *p* < 0.005, Figure 3B] and dentate gyrus [*F*(3,15) = 5.641, *p* < 0.05, Figure 4B]. *Post hoc* analyses revealed that the mTBI alone group was different from all other groups (*p* < 0.05) in both brain regions and was reduced by 42.5% and 22.3% versus sham values in cortex and dentate gyrus, respectively. Correlated with this was an elevation in apoptotic cell number, as revealed from BID staining in the cortex [*F*(3,13) = 23.067, *p* < 0.0001, Figure 3C] and in dentate gyrus [*F*(3,13) = 6.301, *p* < 0.05, Figure 4C]. Likewise, *post hoc* analyses demonstrated that the mTBI group was different from all other groups (*p* < 0.0001, *p* < 0.05, respectively; and 2.76- and 1.91-fold compared to their respective sham values). In addition and illustrated in Figures 5A and 6A, mTBI-challenged mice had an elevation in astrocyte number (3.37- and 1.39-fold, respectively), as revealed by GFAP staining, within the cortex [*F*(3,13) = 37.641, *p* < 0.0001, Figure 5B] and dentate gyrus [*F*(3,13) = 13.284, *p* < 0.001, Figure 6B]. The administration of 3,6′-dithiothalidomide 1 h post injury ameliorated all mTBI-induced changes in neuron, BID, and astrocyte number as, notably, no differences were found between the mTBI + 3,6′-dithiothalidomide and the sham groups. Finally, no changes were evident between any groups (sham, mTBI, and mTBI + drug) in the total cell numbers, as revealed from DAPI staining, within the cortex and dentate gyrus [*F*(3,15) = 1.009, NS, Figure 5C; *F*(3,15) = 2.251, NS, Figure 6C].

In conclusion, the early administration (1 h post injury) of a single dose of the TNF-α synthesis inhibitor 3,6′-dithiothalidomide inhibited cellular changes induced by mTBI in two key brain regions evaluated, cerebral cortex and dentate gyrus.

## Discussion

TNF-α has been implicated in the pathogenesis of a wide number of neurological disorders that develop both acutely, as in TBI and stroke, and chronically, as in Alzheimer’s disease and Parkinson’s disease [29-36,40-42,55,56]. The current study confirms the rapid generation and release of TNF-α in a mouse closed head 50 g weight drop mTBI model, emulating a concussive head injury in humans, which led to neuronal loss and specific cognitive deficits. The inhibition of TNF-α synthesis blocked the mTBI-induced rise in brain TNF-α and protected against neuronal loss and cognitive deficits with a therapeutic window of 12 h. These results underline a role for TNF-α as a key regulator of cascades leading to neuronal loss and cognitive impairment in mTBI and highlights TNF-α as an amenable drug target for future mTBI treatment.

In light of (i) the high incidence of mTBI (approximately 600 per 100,000 people); (ii) the increased risk of dementia resulting from mTBI, particularly in the older people [15]; (iii) the upregulation of pathways leading to chronic neurodegenerative disorders induced by mTBI [12,20,57,58]; (iv) the long-term care, suffering, and economic debt associated with mTBI patients [59]; and (v) the lack of any available therapeutic [60], it is important to understand the mechanisms that underlie head injury.

TNF-α is a well-characterized protein that regulates numerous cellular processes, including inflammation and cell death as well as cellular differentiation and survival, by binding to and activation of two cognate receptors: TNF-α receptor 1 (TNFR1) (p55) and TNFR2 (p75) [29-31,61].

TNFR1 is expressed ubiquitously, including neurons, astrocytes, and microglia throughout the brain. With its intracellular death domain, it contributes to neuronal dysfunction and death and primarily is activated by soluble TNF-α [62]. TNFR2, on the other hand, is principally expressed on hematopoietic cells but also is present on other cell types, including neurons, has been associated with cell survival [61,63-65] and chiefly responds to membrane-bound TNF-α [66,67]. The engagement of homotrimeric TNF-α (either soluble or membrane bound) to either receptor can activate three major signaling pathways: an apoptotic cascade initiated via the TNF-α receptor-associated death domain, a nuclear factor kappa B (NFκB) signaling a pro-survival pathway implemented via NFκB-mediated gene transcriptional actions, and a c-Jun N-terminal kinase (JNK) cascade involved in cellular differentiation and proliferation that is generally proapoptotic [38,68]. In large part, the contrasting pro-survival versus death-induced actions of TNF-α plausibly rely on which TNF-α receptor subtype is activated, the target cell types involved and their expression ratio of TNFR1/2 and associated coupling proteins, and the temporal concentrations of available soluble and membrane-bound TNF-α [64]. However, cross talk between the different signaling pathways and the degree and duration of neuroinflammation combine in determining the eventual physiological consequences of TNF-α receptor activation [69]. Consequent to the diverse actions of TNF-α and the influence of the brain microenvironment in which they occur, it is not always clear under which conditions TNF-α promotes beneficial versus deleterious neuronal effects. This explains the sometimes contradictory literature in the TNF-α neuroscience field [29-31,36,38,55,69] and its involvement in cascades promoting neuronal dysfunction and loss in both acute and long-term neurodegenerative disorders. In the present study, no differences were evident across the sham and mTBI groups in relation to the broad measure of ‘well being’ or in the evaluation of body temperature, anxiety-related behavior, and motor activity, which is in accord with previous results in rodents [51] and humans [70]. Although indistinguishable across a wide number of measures, importantly, deficits in cognitive performance were apparent in mTBI mice in accordance with past studies in mice [24,44-46] and humans [71]. In evaluating potential mechanisms responsible for these cognitive changes, a mTBI-triggered inflammatory cascade mediated by the generation of proinflammatory cytokines appears likely [72]. In this regard, the proinflammatory cytokine TNF-α is considered essential for both initiating and regulating an inflammatory response to trauma, and early transient elevations in brain mRNA expression of TNF-α as well as rises in IL-1β and IL-6 have been described in rodent closed head TBI, and associated adverse events [33,35,73]. In the current study, a time-dependent elevation in brain TNF-α protein levels was apparent in mTBI-challenged mice that peaked at 12 h and declined to baseline by 18 h. In line with this, elevated brain protein levels of TNF-α, IL-1β, and IL-6 have been reported in rodent mTBI models as well as within human CSF within hours of injury [74-78], as they have in other neurological disorders [79-81]. Inhibiting such an elevation in brain TNF-α in this model allowed the evaluation of the role of this transient TNF-α rise in neuronal cell loss, neuroinflammation, and cognitive deficits known to accompany mTBI.

To define the relationship between the mTBI-induced elevation in TNF-α and cognitive impairment evident 7 days later, 3,6′-dithiothalidomide was administered 1, 12, and 18 h following mTBI, extending our initial concentration-dependent studies of the compound in this same mTBI model [44]. Notably, mTBI-induced impairments in both the Y-maze and NOR paradigms were blocked by a single drug dose either at 1 or 12 h post injury, the peak of TNF-α generation in brain, but were not mitigated when administration was delayed to 18 h, thereby defining a treatment window of opportunity.

To evaluate the basis of the mTBI-induced cognitive impairment, brain regions ipsilateral to the side of injury were evaluated at 72 h, as this time coincides with the substantial occurrence of markers of neuronal apoptosis [24,82]. Assessment of the cerebral cortex, the area closest to the site of impact, and dentate gyrus of the hippocampus was performed, as dysfunction in the former and latter might explain the decline in performance in visual memory evaluated by NOR [83] and in spatial learning as assessed by the Y-maze [48], respectively. Neuronal loss (NeuN), an increase neuronal apoptosis (BID), and an elevation in astrocyte number (GFAP) were evident in both brain regions, which is in accord with prior studies in this mTBI model describing elevations in apoptotic proteins (p53, c-Jun, and Bcl-2) as well as TUNEL-positive and silver stain-impregnated degenerating neurons [82,84], as well as other animal models of brain injury [20,60,85,86]. Importantly, early post injury treatment with 3,6′-dithiothalidomide fully prevented these changes. In line with this, this same agent has recently been reported to ameliorate neuroinflammation and alleviate cognitive deficits arising from intracerebral administration of LPS or amyloid-β peptide [79,87,88]. 3,6′-Dithiothalidomide is also reported to attenuate inflammatory markers, Alzheimer’s disease pathology, and behavioral deficits evident in aged Alzheimer transgenic mice [79,80], as well as mitigate neuroinflammation and apoptosis within the penumbra of focal ischemic stroke in mice [81]. Additionally, 3,6′-dithiothalidomide has recently been described to lower TNF-α and cerebral aneurysm formation and progression to rupture in mice [89,90].

Taken together, these studies support an important role for TNF-α in neuroinflammation and the modulation of neuronal function and viability across a broad range of neurological disorders. Consequent to the availability of both biological and small molecular weight TNF-α inhibitors in preclinical and clinical research, there is growing evidence that whereas physiological TNF-α levels are critical in normal brain physiology [37,38,55], excess TNF-α plays a key role in brain dysfunction [29-31,69]. In relation to the former, among a host of functions in brain, TNF-α serves as a gliotransmitter that, when secreted from glial cells surrounding synapses, can regulate synaptic communication between neurons as well as neuronal networks [36-38]. With respect to the latter, TNF-α reductions achieved with the clinical TNF-α binding protein etanercept, when administered i.p. following fluid percussion injury-induced TBI, attenuated TBI-induced contusion, ischemia, and resulting motor and cognitive deficits [91]. As in our studies, this brain TNF-α lowering approach also mitigated TBI-induced elevations in [91]. Albeit that these animal studies utilized a far higher etanercept dose than achievable in humans [91], in an open-label analysis of 12 TBI patients given perispinal etanercept up to more than 10 years following injury, motor impairment and spasticity were reported significantly reduced [55], supporting both clinical and translational relevance. Additionally, in rat studies using a TBI weight drop paradigm with some parallels to our studies in mice, immediate i.v. administration of a TNF-α binding protein or the competitive nonselective phosphodiesterase inhibitor, pentoxifylline, that lowers TNF-α at a transcriptional level, has been reported to mitigate mTBI-induced brain edema at 24 h and neurological dysfunction evaluated up to 14 days [75]. Finally, in other animal models that include ischemic and spinal cord injury, thalidomide has been reported to effectively reduce inflammation and improve the injury outcome when administered either before [92], immediately after [93], or within an hour of injury [94] in doses that varied between 20 and 300 mg/kg.

The single dose of 3,6′-dithiothalidomide used in the current study (28 mg/kg in mouse) compares favorably with former studies of thalidomide (20 to 300 mg/kg) and equates to a dose of 150 mg in a 65 kg human (2.3 mg/kg), following normalization of body surface area in accord with FDA guidelines [95]. Prior cellular [43,79] and animal studies [80,81] indicate that 3,6′-dithiothalidomide (albeit administered systemically by the i.p. route, as in the current study) is more potent in reducing TNF-α elevations than thalidomide and that it enters the brain but does not appear to be soporific [44,79,80]. In light of recent studies suggesting that thalidomide analogues can express more potent anti-inflammatory action with less neurotoxicity than the parent compound [96,97], the development of new and well-tolerated small molecular weight TNF-α inhibitors that can be administered orally may be of great clinical potential. Past studies evaluating genetically engineered mice that either lack TNF-α or its receptors have suggested a ‘Jekyll and Hyde’ scenario in which elevated TNF-α is detrimental during the acute phase after a TBI incident, but a part of the regenerative processes during the later chronic post-injury phase [42,98,99]. More recent studies in which the two individual receptors, TNFR1 (p55) and TNFR2 (p75), have been separately deleted suggest that each may have a distinct time-dependent function in TBI [40,41]. TNFR1 knockout mice possessed a smaller contusion volume and a clearly improved neurobehavioral performance for up to 4 weeks following a controlled cortical impact TBI, as compared with wild-type mice, whereas TNFR2 knockout mice demonstrated significant worsening post injury [42]. This implicates TNFR1 involvement in the immediate deleterious actions associated with acute TNF-α release following an injury and an involvement of TNFR2 in later tissue repair.

In summary, our studies suggest that the administration of a TNF-α synthesis inhibitor, 3,6′-dithiothalidomide, within the initial 12-h window of a mTBi event, may be therapeutically valuable at a time when elevated TNF-α interacts with TNFR1 to drive the development of neuroinflammation, neuronal dysfunction, and apoptosis. But such a therapeutic strategy should best be acute to allow later potentially beneficial actions of TNF-α mediated via TNFR2. Our results, together with other studies [33,36,39-41,44,56,74-78,97], underscore the potential of TNF-α as a potential therapeutic target in TBI and other neurological disorders.

## Conclusion

This study implicates TNF-α in the delayed neuronal cell death and gliosis that occurs within the brain following mTBI, which leads to cognitive deficits. It additionally indicates that pharmacologically limiting the elevation of TNF-α within 12 h of the mTBI event markedly reduces such secondary damage and leads to improved cognitive outcome measures. Such a window provides an opportunity for translational studies in mTBI that is more difficult to define for other neurological disorders [100]. Building on the growing literature on the role of TNF-α in the initiation and perpetuation of the neuroinflammation that can drive the progression of acute and chronic neurological disorders [29-31,36,55,56,68,69,101,102], the present study underscores the value of targeting of TNF-α as a treatment strategy for TBI and the development of new and well-tolerated oral small molecular weight TNF-α inhibitors and related approaches as clinical treatment options.
